# VitroGel-loaded human MenSCs promote endometrial regeneration and fertility restoration

**DOI:** 10.3389/fbioe.2023.1310149

**Published:** 2024-01-08

**Authors:** Meijuan Wu, Shengnan Wu, Shidong Tan, Qingxin Xu, Donghai Zhang, Jiaxue Sun, Haoyu Yang, Cancan Wang, Tao Duan, Yao Xu, Zhiyun Wei

**Affiliations:** Shanghai Key Laboratory of Maternal Fetal Medicine, Shanghai Institute of Maternal-Fetal Medicine and Gynecologic Oncology, Clinical and Translational Research Center, Shanghai First Maternity and Infant Hospital, School of Medicine, Tongji University, Shanghai, China

**Keywords:** intrauterine adhesions, rat model, MenSCs, hydrogel, fertility, cell therapy

## Abstract

**Introduction:** Intrauterine adhesions (IUA), also known as Asherman's syndrome, is caused by trauma to the pregnant or non-pregnant uterus, which leads to damaged endometrial basal lining and partial or total occlusion of the uterine chambers, resulting in abnormal menstruation, infertility, or recurrent miscarriage. The essence of this syndrome is endometrial fibrosis. And there is no effective treatment for IUA to stimulate endometrial regeneration currently. Recently, menstrual blood-derived stem cells (MenSCs) have been proved to hold therapeutic promise in various diseases, such as myocardial infarction, stroke, diabetes, and liver cirrhosis.

**Methods:** In this study, we examined the effects of MenSCs on the repair of uterine adhesions in a rat model, and more importantly, promoted such therapeutic effects via a xeno-free VitroGel MMP carrier.

**Results:** This combined treatment reduced the expression of inflammatory factors, increased the expression of anti-inflammatory factors, restricted the area of endometrial fibrosis, diminished uterine adhesions, and partially restored fertility, showing stronger effectiveness than each component alone and almost resembling the sham group.

**Discussion:** Our findings suggest a highly promising strategy for IUA treatment.

## 1 Introduction

Female infertility, affecting approximately 9%–18% of women worldwide, is predicted by the World Health Organization to be the third most common disease by the end of the 21st century, after cancer and cardiovascular disorders. A stable uterine environment is essential for embryo implantation and development in a successful pregnancy. About 8% of infertility cases are secondary to intrauterine adhesions (IUA) (Evans-Hoeker and Young, 2014). IUA means that fibrous tissue forms inside the uterus and can lead to the walls of the uterus sticking together, which fully or partially closes off the cervix and/or the uterus cavity, resulting in pelvic pain and infertility (Hooker et al., 2014). IUA can be caused by several obstetric and gynecological diseases, including placental retention (Hamerlynck et al., 2013), endometrial fibroplasia (Xin et al., 2022), and endometrial damage, as well as many common surgeries, such as miscarriage clearance (Hooker et al., 2014), myomectomy (Mazzon et al., 2014), polypectomy (Perez-Medina et al., 2005) and insertion of intrauterine devices (IUDs) (Pabuccu et al., 2008). IUA accounts for almost 90% of reproductive disorders (Deans and Abbott, 2010; Evans-Hoeker and Young, 2014). Reducing endometrial tolerance, IUA has been linked to recurrent miscarriage and is thought to be the cause in 20%–30% of these cases (Hooker et al., 2014). Hysteroscopy is currently an effective tool for IUA diagnosis (Kuroda et al., 2022), and is capable of separating mild adhesions to some extent. However, separation is difficult for severe adhesions, followed with worse clinical outcomes. Even if the separation is successful, recurrence is frequent and unpredictable without available prevention approach, especially when sufficient post-operation nursing care is not taken. Novel strategies are thus urgent to be developed for effective and long-lasting treatment against IUA.

Stem cell therapy is currently one of the research hotspots in endometrial regeneration. Transplantation of mesenchymal stem cells (MSCs) is widely used for both damage repair and disease treatment (Aurich et al., 2007). Among various MSCs, menstrual blood-derived mesenchymal stem cells (MenSCs) are highlighted by their non-invasive source. MenSCs have a high rate of growth and self-renewal, share many of the characteristics as MSCs, including therapeutic applications, broad availability, and excellent biocompatibility, and therefore offer a promising new avenue for research in stem cell regenerative medicine (Gu et al., 2015). MenSCs have been found to be useful in treating diseases, including ovarian failure, autoimmune diseases, diabetes, and inflammatory response (Zhang et al., 2021; Li et al., 2022; Mirzadegan et al., 2022). Nevertheless, MenSCs, as well as other MSCs, have been observed to be inconstant in treatment efficacy, probably due to their uncontrollable periods of stay in the lesion and highly variable local environments (Galipeau and Sensebe, 2018; Wechsler et al., 2021).

VitroGel MMP is a xeno-free hydrogel system that has been enhanced on biocompatibility and biodegradability with matrix metalloproteinase (MMP)-sensitive peptides. Designed for providing optimized environment for cell growth, it supports various biological activities, such as cell proliferation, migration, differentiation, angiogenesis, and apoptosis. More importantly, hydrogels have wild clinical potentials. Being developed as carrier of MSC treatments, it has been reported to promote the functional and structural recovery of colitis (Cao et al., 2020), to regulate neuronal differentiation and suppress inflammatory reaction (He et al., 2021), to limit secondary injury after traumatic brain injury (Alvarado-Velez et al., 2021), to enhance vascularized sweat gland regeneration, to promote craniofacial bone regeneration (Hasani-Sadrabadi et al., 2020), as well as to increase functional osteochondral regeneration and chondrogenesis (Kwon et al., 2018). In this study, we examine whether VitroGel MMP could support and improve MenSCs to inhibit IUA and promote fertility in a rat model, and find the effectiveness of this combined treatment.

## 2 Materials and methods

### 2.1 Isolation and culture of MenSCs

Three healthy women who were experiencing their periods voluntarily donated their menstrual blood to Shanghai First Maternity and Infant Hospital. All of the above volunteers were between the ages of 30–35, with BMI ranging 18.5–24, had regular menstruation, and had no history of gynecological, cardiovascular, respiratory, neurological, immune, digestive or endocrine diseases. Before the start of the study, written informed consent was provided by each donor. The study was proved by the ethical committee of Shanghai First Maternity and Infant Hospital affiliated with Tongji University School of Medicine. The menstrual blood samples were taken for 3 h with menstrual cups (Diva International, Inc., Canada) on day 2 and/or 3 of the menstrual cycle. Mononuclear cells were separated using Ficoll-Paque (Cytiva, US) within 0.5 h after collection. The harvest fraction was then gently transferred into an equal volume of phosphate-buffered saline (PBS) containing 0.25 mg/mL amphotericin B, 100 U/mL penicillin, 100 mg/mL streptomycin, and 2 mM ethylenediaminetetraacetic acid (EDTA) (Cytiva). After centrifuging at 100 g, room temperature for 10 min, and washed with PBS twice, the cell pellet was generally suspended in MenSC culture medium (E-vans Biotech, China). Cells were then seeded into a culture flask and cultivated in a humid incubator at 37°C with 5% CO_2_. Cells were washed by PBS to discard the non-adherent cells after 2 days of incubation. Every 3–4 days, the medium was renewed until adherent cells reached 80%–90% confluency (P0), at which point cells were dissociated with 0.25% trypsin-EDTA (NCM Biotech, China) and seeded into new flasks with a 1:4 ratio. Passage of MenSCs was tracked during cultivation.

### 2.2 Flow cytometry

MenSCs were dissociated with 0.25% trypsin-EDTA and suspended in the staining solution (PBS). A total of 3 × 10^6^ MenSCs were incubated for 30 min in the dark at 4°C with each antibody according to the manufacturer’s instructions: APC anti-human CD73 (Cat. No. 344005; BioLegend, US), APC anti-human CD90 (Cat. No. 328113; BioLegend), APC anti-CD105 (Cat. No. 800507; BioLegend), PE anti-human CD276 (Cat. No. 331606; BioLegend), APC anti-human HLA-A, B, C (Cat. No. 311409; BioLegend), FITC Mouse Anti-Human CD34 (Cat. No. 560942; BD Pharmingen, US), APC anti-human CD44 (Cat. No. 338805; BioLegend) and FITC Mouse Anti-Human HLA-DR (Cat. No. 560944; BD Pharmingen). The cells were then acquired in a BD FACSCalibur flow cytometer (BD Biosciences, US) and analyzed using the FlowJo software (version 10.8.1).

### 2.3 ELISA assay

On the third day after the MenSCs were seeded at passage 5 with the full culture medium containing fetal bovine serum, when the cell density was approximately 70%–80%, the conditioned medium was collected and spin down at 12,000 g, room temperature for 15 min. The cell-free supernatant was used in the ELISA assay. The fresh full culture medium was used as the negative control. ELISA assay was performed following the manufacturer’s instructions. Target-specific ELISA kits were purchased from WELLBIO (China) for human VEGF (Cat. No. EH6532S), human TGF-β1 (Cat. No. EH6481S), human PDGF-BB (Cat. No. EH6403S), and human MMP-3 (Cat. No. EH6370S). OD450 values were measured with a microplate reader (Thermo Fisher Scientific, US).

### 2.4 Rat model of IUA

SD rats (150–200 g, 6–8 weeks old) were purchased from Charles River (US) and were raised in a pathogen-free environment with a constant temperature of 23°C ± 2°C, a light-dark cycle of 12 h, and a relative humidity of 50%. The rats had free access to water and standard rat chow, and they were observed for a full week before the experiments began to make sure they were in good health.

The estrous cycle of female rat was determined by flushing vagina with sterile saline at 7:30 a.m., drying 50 uL lavage on the glass slide, and staining with Wright and Giemsa solution (Servicebio, China). Female rats were randomly assigned into five groups after two estrous cycles (around 8 days): Sham, PBS, MenSCs, VitroGel, and MenSCs + VitroGel. To establish the IUA model, rats were injected at the gluteus maximus for general anesthesia with Zoletil 50 (tiletamine hydrochloride: zolazepam hydrochloride = 1 : 1; 40 mg/kg; Virbac Laboratory, France) after fasting for 12 h, their belly cavities were opened and uteri was exposed, and then the left horns of the uteri were scratched with a 20-gauge syringe until the uterine walls became congested and rough to establish IUA pathology for all rats, except for the Sham group in which the abdomens were cut open without scratching the uteri. One week later, we slowly injected 200 ul PBS, 1 × 10^5^ MenSC cells (P5) suspended in 200 uL PBS, 200 uL VitroGel MMP solution, and 1 × 10^5^ MenSC cells (P5) suspended in 200 uL VitroGel MMP solution with 1 mL syringes into the left horns of the uteri after opening belly cavities, for the PBS group, the MenSCs group, the VitroGel group, and the MenSCs + VitroGel group, respectively. Rats were euthanized with CO_2_ inhalation. All animal studies and euthanasia procedures were approved by Tongji University’s Animal Care Committee (protocol code TJBG02022201).

### 2.5 Histological analysis

Rat uteri tissue was collected and fixed with 4% paraformaldehyde (Sangon, China), then dehydrated, and embedded in paraffin. A series of 4-μm sections were prepared and two of every five sections were selected for hematoxylin and eosin staining (H&E) and Masson staining. The slides were evaluated under the light microscope (E100, Nikon, Japan). The total number of vessels and glands were counted and the fibrosis condition was evaluated.

### 2.6 Immunohistochemistry

Paraffin sections were dewaxed with a dewaxing solution and rehydrated with anhydrous ethanol before immunohistochemistry. Following high-temperature and high-pressure antigen retrieval, the sections were treated with 3% H_2_O_2_ at room temperature in the dark for 25 min to inhibit endogenous peroxidase activity, and then washed with PBS for 3 times. Subsequently, the sections were incubated with 3% BSA at room temperature for 30 min. After the serum blocking buffer was shaken off gently, the sections were incubated with the primary antibodies (TGF-β1 Rabbit pAb, A15103, ABclonal, China; PDGFβ Rabbit pAb, A1195, ABclonal) at a dilution of 1:200 at 4°C overnight. On the next day, all sections were washed with PBS and incubated with the secondary antibody (GB21303, Servicebio) for 50 min at room temperature, and then stained using 3, 3′-diaminobenzidine (DAB). Finally, the sections were counterstained with hematoxylin for 3 min and washed with PBS. The positive signals were observed and evaluated by an optical microscope (E100, Nikon), and the IOD/area (Integrated Optical Density) and percent positive rate were analyzed by Image Pro Plus 6.0 software.

### 2.7 RT-qPCR

Total RNA was isolated from rat uterine tissue using the RNAiso Plus Reagent (Takara, Japan) following the manufacture’s instruction, and quantified using a NanoDrop 2000c Spectrophotometer (Thermo Fisher Scientific). Reverse transcription was performed using the Evo M-MLV RT Premix kit (Accurate Biotechnology, China), and cDNA was input into qPCR system with the SYBR Green Pro Taq HS Premix Kit (Accurate Biotechnology). Reaction of qPCR was run in technical duplicates on an ABI ViiA 7 platform (Thermo Fisher Scientific). The primer sequences for RT-qPCR are shown in Table 1. Specificity of qPCR reaction was confirmed by single peak in melting curve. The relative expression of each gene was normalized to GAPDH.

**TABLE 1:** Sequences of primers used in RT-qPCR.

Gene	Forward primer 5′→3′	Reverse primer 5′→3′
collagen I	ATGTTCAGCTTTGTGGAC	GAG​ATG​ATG​CTT​TGA​CAG​ATG
HB-EGF	TCT​GTC​TGT​CTT​CTT​GTC​AT	TTC​CAA​GTC​ATA​ACC​TCC​TC
IL2	TGA​GTG​CCA​ATT​CGA​TGA​T	GAG​ATG​ATG​CTT​TGA​CAG​ATG
Lif	CCCTACTGCTCATTCTGC	AGT​TGA​CTC​TTG​ATC​TGG​TT
αvβ3	CATCTGTACCACGAGAGG	AGACTCATCTGAGCACCA
IL10	GTAGCCACCCAACAAACA	GAG​ACA​GAC​AAG​CAA​GAG​AT

### 2.8 Fertility test

After mating one female with one male rats, the day 0.5 of gestation was determined when sperms were observed in vaginal smears. Then the number of embryo implantation was recorded at 16.5 days of gestation. No structural abnormalities of the fetus and placenta were observed (fetus with no eyes, no face, few fingers or deformity, no anus, and placenta with hypertrophy, atrophy, and necrosis). The rate of embryo implantation was calculated by dividing embryo count on left horn by total embryo count on both horns.

### 2.9 *In vivo* imaging

MenSCs were labeled with DiR iodide (Cat. No.22070, AAT Bioquest, US) before uterine transplantation. 3 × 10^5^ cells were incubated in 5 μM DiR solution at 37°C for 20 min, then washed twice with PBS. Next, the DiR-labeled MenSCs were transplanted into rat uterus. The image capture and analysis were done by an *in vivo* imaging system (Tanon, China) at day 0, day 4 and day 8.

### 2.10 Statistical analysis

The mean values of experiments performed in triplicate are expressed as the mean ± SD. Statistical analysis of the results of all data were performed using GraphPad Prism 9 software to perform Student’s t-test or one-way analysis of variance (ANOVA) followed by Fisher’s LSD test, after confirming normal distribution using K-S test. Significance was defined as *p < 0.05, **p < 0.01, ***p < 0.001.

## 3 Results

### 3.1 Verification of primary MenSCs

**FIGURE 1:**
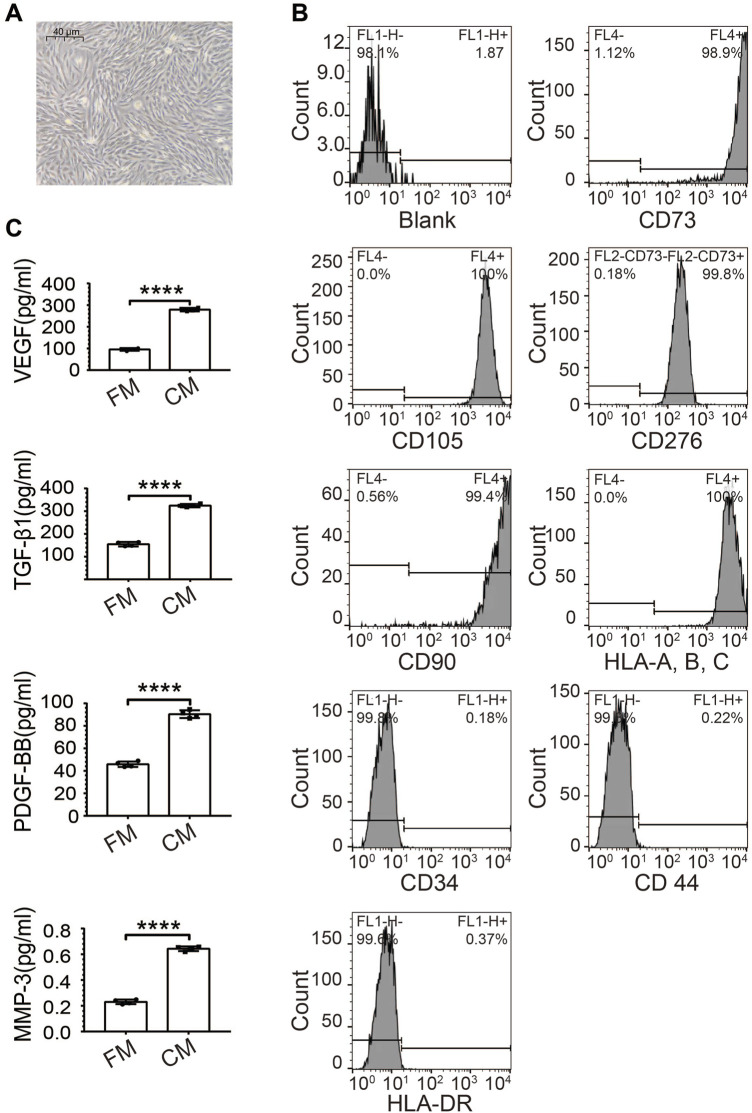
Characteristics of human MenSCs. **(A)** Spindle-shaped morphology of isolated MenSCs at passage 5 under the microscope (40 um). **(B)** Verification of MenSCs by flow cytometry with both positive (CD73, CD90, CD105, CD276, and HLA-A, B, C), and negative surface markers (CD34, CD44, and HLA-DR). **(C)** Secreted stem cell factors quantified by ELISA, including PDGF-β1, TGF-β1, VEGF, and MMP3. Blank: Cell suspensions without antibody. N = 4; ****, *p* < 0.0001; *t*-test. FM, fresh medium; CM, conditioned medium.

We isolated MenSCs from human menstrual blood and kept their primary culture at low passages (up to 24). MenSCs were firstly verified by light microscope for their typical fibroblast-like morphology (Figure 1A) (Tan et al., 2016). Molecular Identification of MenSCs was performed upon the 5th passage using a broad range of surface markers by flow cytometry, based on summarization of various previous studies (Alcayaga-Miranda et al., 2015). More than 98.9% of cultured MenSCs were positive with stem cell markers, including CD73, CD90, CD105, CD276, and HLA-A, B, C (Figure 1B). Moreover, the cultured MenSCs were also negative on CD34, CD44, and HLA-DR signals for discrimination from hematopoietic stem cells, white blood cells, and activated T cells, respectively (Figure 1B). Mesenchymal stem cells are known to secret diverse factors supporting their functions (Uccelli et al., 2008). Consistently, ELISA assays on conditioned medium of MenSCs quantified strong secretion of PDGF-β1, TGF-β1, VEGF, and MMP3 (Figure 1C). All these observations confirmed the identity of MenSCs.

### 3.2 The combination of MenSCs and VitroGel rebuilt the histological structure of uterus in IUA rat model

**FIGURE 2:**
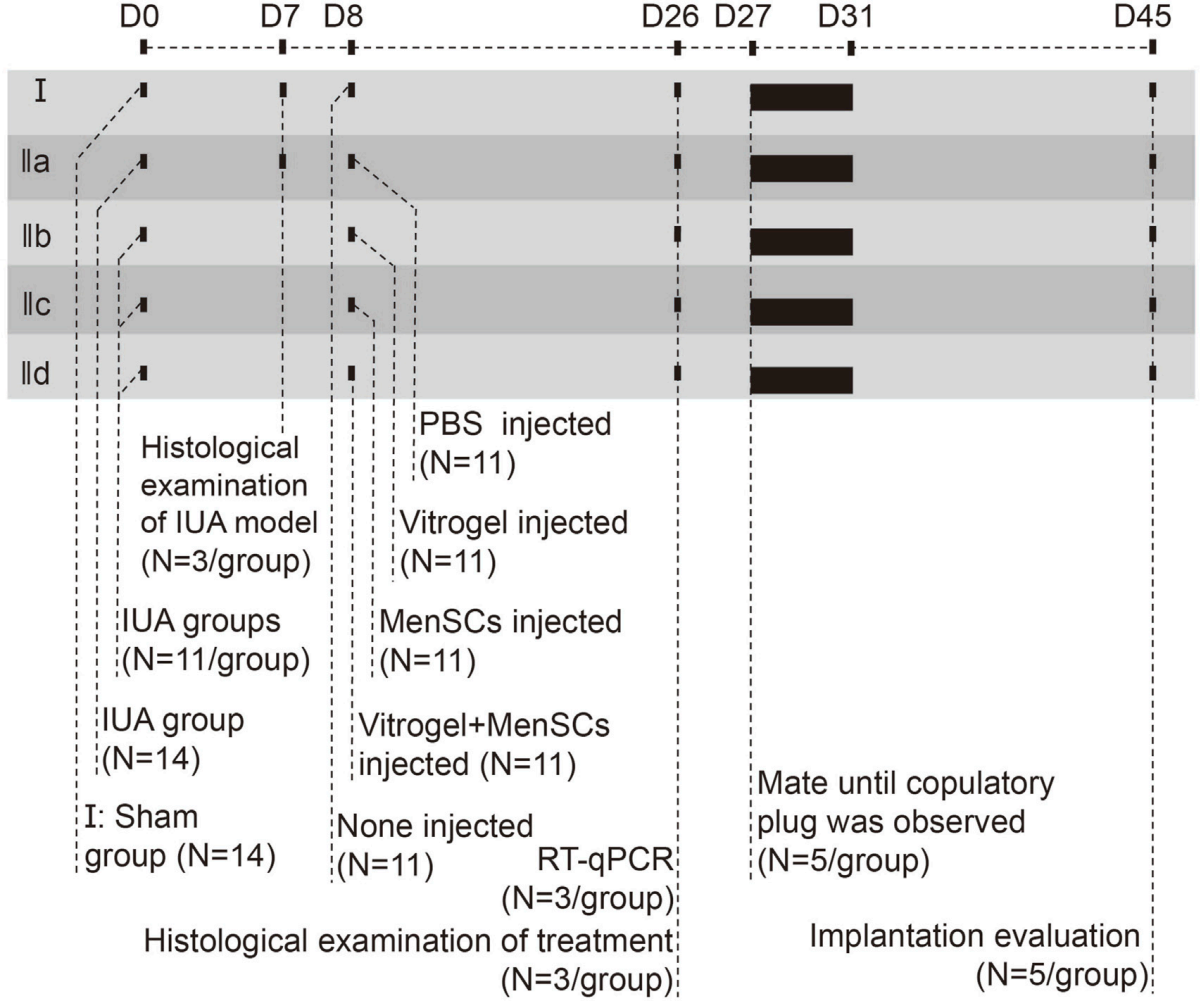
The workflow of the rat experiment.

**FIGURE 3:**
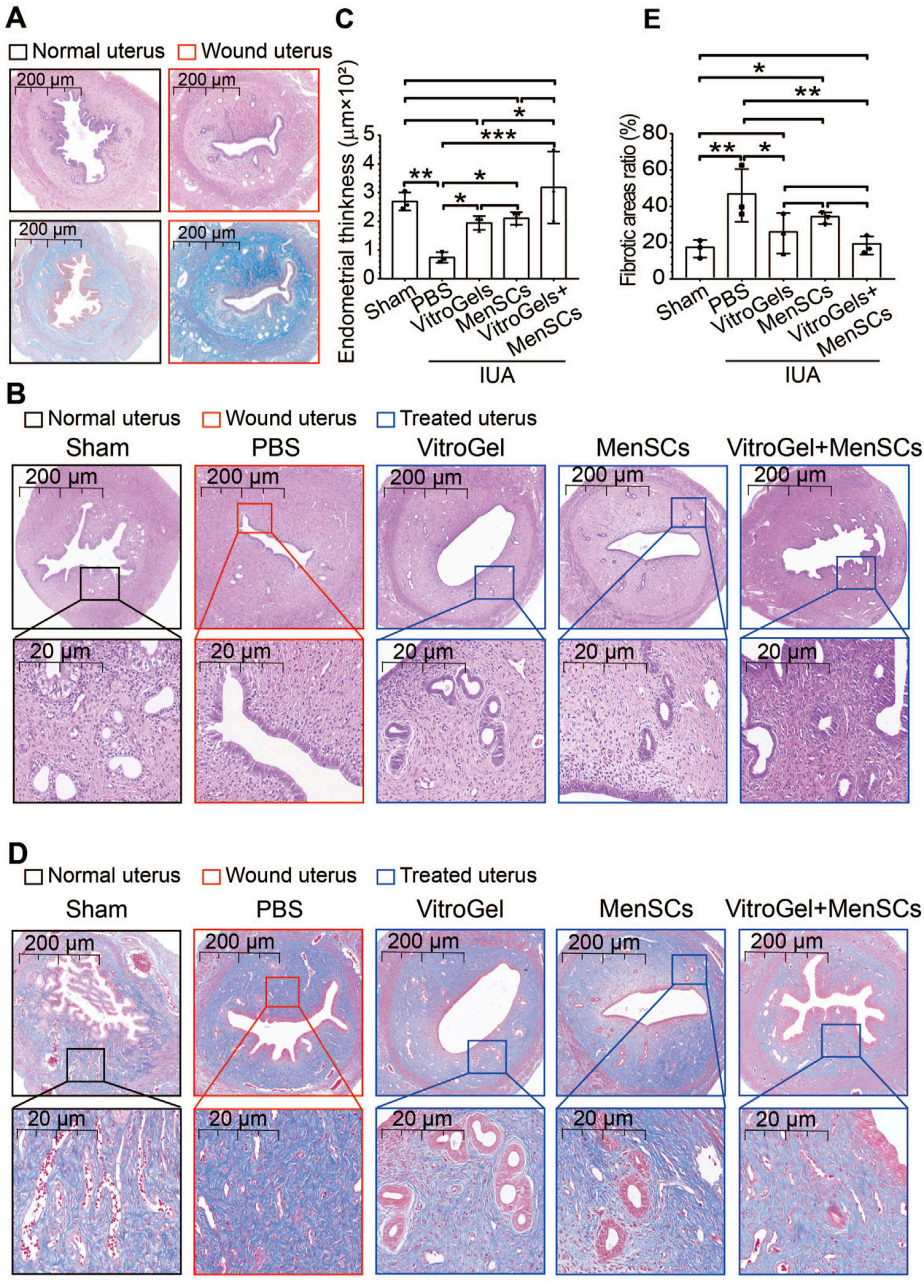
Histological examination of uteruses in rat IUA model with differential treatments. **(A)** H&E and Masson staining of rat uteruses with or without IUA wound on Day 7. **(B)** Representative H&E staining of rat uteruses after various treatments on Day 26. **(C)** Quantified endometrial thickness based on H&E staining (N = 3). **(D)** Representative Masson staining of rat uteruses after various treatments on Day 26. **(E)** Quantified fibrotic area ratios based on Masson staining (N = 3). *, *p* < 0.05; **, *p* < 0.01; ***, *p* < 0.001; ANOVA followed by Fisher LSD test.

The workflow of our rat experiment is illustrated in Figure 2. After random assignment (on D0), female rats were either wounded by uterine scraping (group II) or treated with a sham procedure (group I). Seven days later, six random rats from groups I and II (N = 3 vs. 3) were dissected to ensure the establishment of the IUA model. *In vivo* imaging analysis showed that MenSCs stayed within uteri and decreased gradually (Supplementary Figure S1). H&E staining demonstrated that discontinuous luminal surface, damaged uteri, and loss of the luminal cavity (Figure 3A). Masson staining further showed increased fibrosis in the wounded uterus (Figure 3A). On D8, rats in group II were randomly assigned to four subgroups for different treatments (Figure 2). Eighteen days later, rats with control treatment (PBS) still had IUA histology: narrow uterine cavity, endothelial damage (Figures 3B, C), and widespread fibrosis (Figures 3D, E). Both VitroGel and MenSCs individually increased endometrial thickness significantly comparing to PBS treatment, yet their combination (VitroGel + MenSCs group) further thickened endometrium to a level even comparable to the sham group (Figures 3B, C). Meanwhile, although each of VitroGel and MenSCs reduced fibrotic areas mildly and non-significantly, VitroGel-supported MenSCs diminished fibrosis more strongly with statistical significance comparing to the PBS group (Figures 3D, E).

### 3.3 IUA was repaired via anti-inflammation and angiogenesis

**FIGURE 4:**
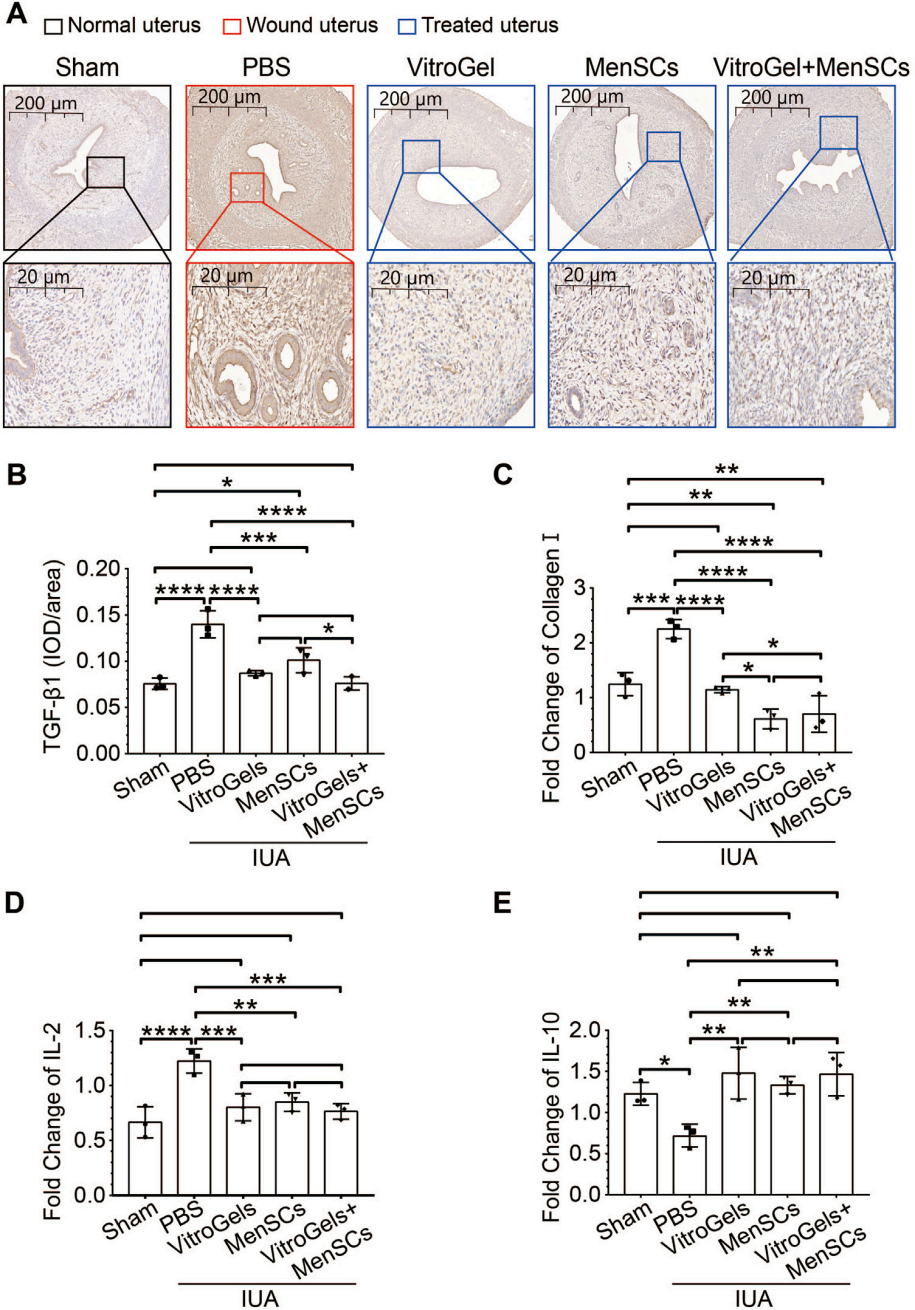
Molecular characteristics of IUA damages in rat IUA model with differential treatments on Day 26. **(A,B)** Representative immunohistochemistry **(A)** and quantification **(B)** of TGF-β1 in uteruses (N = 3). **(C)** RNA level of collagen I in uteruses (N = 3). **(D,E)** RNA level of IL2 and IL10 in uteruses (N = 3). *, *p* < 0.05; **, *p* < 0.01; ***, *p* < 0.001; ANOVA followed by Fisher LSD test.

**FIGURE 5:**
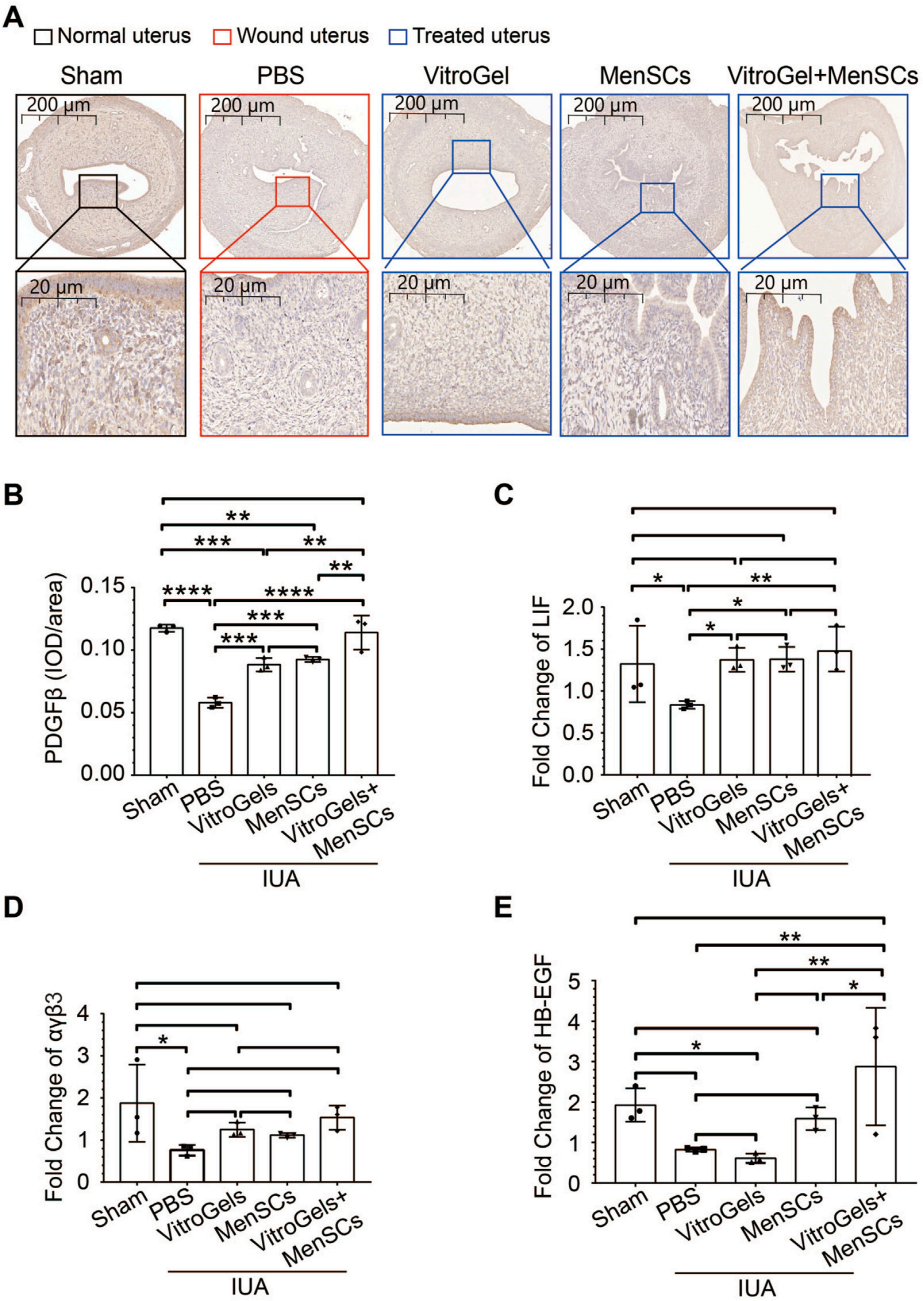
Molecular characteristics of endometrial recovery in rat IUA model with differential treatments on Day 26. **(A,B)** Representative immunohistochemistry **(A)** and quantification **(B)** of PDGFβ in uteruses (N = 3). **(C–E)** RNA level of Lif, αβγ3 and HB-EGF in uteruses (N = 3). *, *p* < 0.05; **, *p* < 0.01; ***, *p* < 0.001; ANOVA followed by Fisher LSD test.

TGF-β1 is a crucial driver of fibrosis (Su et al., 2020). Its protein level was lowered by VitroGel and MenSCs (Figures 4A, B), suggesting the retard of fibrosis initiation. In agreement with Masson staining, RNA expression of collagen I confirmed, at the molecular level, that VitroGel and MenSCs interrupted fibrosis progression significantly (Figure 4C). Besides, inflammatory IL2 and anti-inflammatory IL10 were down- and upregulated by VitroGel and MenSCs comparing to the PBS group, respectively (Figures 4D, E). These observations suggested that the combination of MenSCs and VitroGel alleviated the IUA damage by creating an anti-inflammatory environment and halting the fibrosis process. Moreover, VitroGel and MenSCs, and more significantly their combination, rebuilt the endometrium via angiogenesis (Figures 5A, B) and, more importantly, recovered expression of several markers for endometrial receptivity, including Lif, αβγ3, and HB-EGF (Figures 5C–E). Such recoveries suggested functional endometrial repair by combining MenSCs and VitroGel.

### 3.4 VitroGel-supported MenSCs restored fertility of IUA rats

**FIGURE 6:**
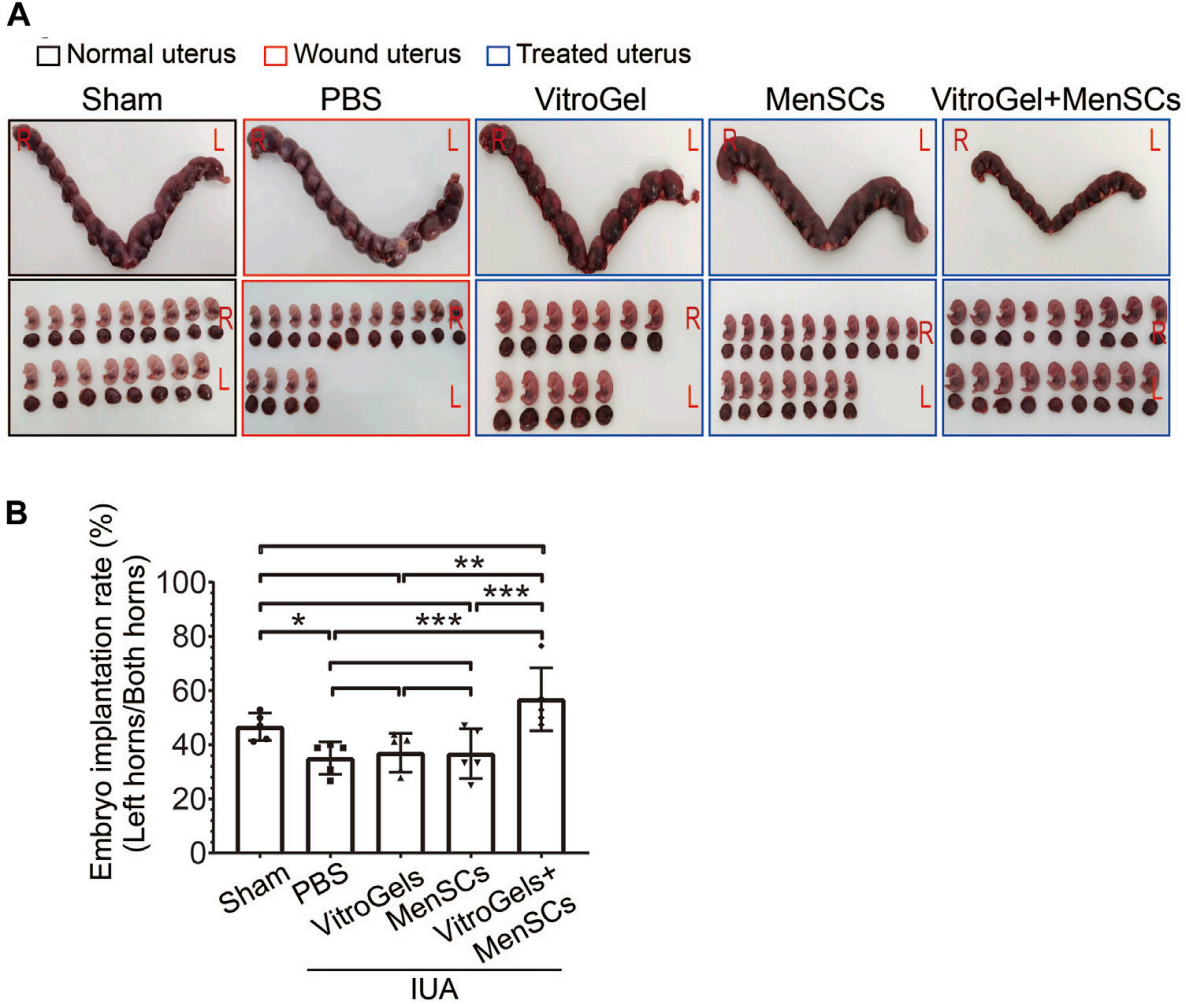
Comparison of fertility after differential treatments in rat IUA model. **(A)** Representative photos of rat uteruses and fetuses. IUA wounds and treatments were performed only on the left sides of uteruses, while the right sides without any operation were used to rule out confounding pregnancy factors. **(B)** Rate of embryo implantation on the left sides of uteruses (N = 5). L = Left; R = Right; rate = embryo count on left horn/total embryo count on both horns. **, *p* < 0.01; ***, *p* < 0.001; ANOVA followed by Fisher LSD test.

Infertility is one of the most devastating symptoms for IUA patients. Indeed, IUA compromised fertility on the rat model, as evidenced by a reduced implantation rate of embryo in the performed left horns of the uterus, comparing the PBS group with the sham group (Figures 6A, B). Although MenSCs and VitroGel could individually repair IUA damages on various histological and molecular characteristics (Figures 2–5), neither significantly increased rate of embryo implantation, comparing to PBS group (Figures 6A, B). To be noted, the combination of MenSCs and VitroGel fully recovered the fertility of IUA rats with significant increase than PBS group, showing a highly promising treatment strategy with strong clinical relevance (Figures 6A, B).

## 4 Discussion

Several clinical and animal researches have revealed that hydrogels and MSCs are safe in treating various diseases (Chaudhuri et al., 2016; Li et al., 2019; Hasani-Sadrabadi et al., 2020; Wang et al., 2020; Zheng et al., 2020; Chen et al., 2022; Tang et al., 2022). In this study, we demonstrated that the application of MenSCs or VitroGel alleviated adhesions to some extent in a rat model of IUA, characterized by the reduced endometrial fibrosis, increased endometrial tolerance, and weakened inflammatory response compared to the PBS group, while the combination of MenSCs and VitroGel was more effective, and recovered the upmost important function of uterus - embryo implantation, which is consistent with existing studies (Liu et al., 2019; He et al., 2022; Xin et al., 2022). These results suggest that MenSCs combined with VitroGel might have a functional therapeutic effect against endometrial damage.

Affecting approximately 1.5%–21.5% of women worldwide, IUA is induced by a variety of endometrial damages, including endometrial inflammation, uterine surgery, spontaneous abortion and retained products of conception. During the healing process, the contralateral uterine wall may adhere, causing partial or total occlusion of the cervical canal and uterine chamber, leading to atypical menstruation, infertility, repeated miscarriages (Yu et al., 2008), or other serious gynaecological issues (Wang et al., 2022). A series of strategies have been developed to prevent and treat IUA, such as microneedle patches (Liu et al., 2019; Gomaa et al., 2021; Zhang et al., 2022), uterine cavity balloons (Chen and Xie, 2016), transcervical resection of adhesion (Deans and Abbott, 2010), and intrauterine scaffolds (Ebrahim et al., 2018; Cai et al., 2019; Ji et al., 2020; Li et al., 2020). With certain level of therapeutic effects, these techniques, however, are still facing many challenges, including high postoperative recurrence rates, frequent dislocation, and biological incompatibility.

MSCs are a type of pluripotent stem cells with ability of self-renewal and multidirectional differentiation, which were initially uncovered in 1976 (Wan et al., 2006) in the bone marrow, and first proposed as a cell therapy in 1995 (Lazarus et al., 1995). A dozen of MSC treatments have been approved thus far, such as the Temcell against acute graft-versus-host disease, and the Alofisel for the treatment of Crohn’s related enterocutaneous fistular disease (Phinney et al., 2013). MSC therapies for many other clinical applications are also in progress, including acute myocardial infarction, acute ischemic stroke, acute tissue injury syndromes, as well as chronic degenerative and inflammatory diseases (Hare et al., 2012; Gao et al., 2015) MSCs can be isolated from a variety of tissues or body fluids. However, MSCs derived from adipose tissue or umbilical cord blood have frequent rejection and ethical restrictions, while bone marrow MSCs often have pain and infection problems. MenSCs are a novel provenance of MSCs isolated from menstrual fluid (Khoury et al., 2014). MenSCs are simple to obtain non-invasively, have low immunogenicity, and can be autologously transplanted. Therefore, MenSCs are more suitable source of MSCs for stem cell therapy, and consistently with previous reports (Zheng et al., 2018; Chang et al., 2020), show ability to help repair endometrial damage and alleviate symptoms of IUA in a rat model. Beyond using MenSCs directly, a recent study used MenSCs-derived exosomes and found prevention of endometrial fibrosis in IUA model via modulating YAP ubiquitination (Zhang et al., 2019; Qi et al., 2023).

Adhesion and retention at the application site as well as the regenerative properties of the MSCs are vital factors for successful tissue repairing and regeneration. Therefore, various hydrogels are being developed to deliver stem cells in diverse clinical applications due to their mechanical properties (Qiao et al., 2021; Han et al., 2022; Tang et al., 2022; Jirigala et al., 2023). We used a new material, VitroGel, modified with MMP-sensitive peptides, in this study with the balanced considerations that it supports MenSCs for enough period to allow functioning while does not last too long so as to avoid any biosafety concerns. MMPs, secreted by MenSCs (Figure 1C), were proved to contribute in various biological processes, such as tissue growth and remodelling, trauma repair, tissue defence mechanisms, and immune response (Lindsey, 2018; Zinter et al., 2019; Shen et al., 2021; de Almeida et al., 2022). In combination with MenSCs for treating IUA in rats, which has not been reported so far, the tunability of the hydrogel creates an optimized microenvironment for cell growth and thus enhances the repairment function of MenSCs. Although the detailed molecular mechanism on interaction between VitroGel and MenSCs is not well depicted, many studies consider biosupport, rather than cell signalling, as the major role of hydrogel (Wisdom et al., 2018; Jin et al., 2023; Xu et al., 2023).

We further explored the underlying mechanism of MenSC effectiveness on treating IUA. Type 1 collagen (Collagen I) is the most abundant collagen in many human tissues, such as bone, skin and tendons. Collagen I expression levels are regulated by multiple mechanisms, and Collagen I overexpression is positively associated with tissue fibrosis diseases. It has also been documented that Collagen I contributes to fibrosis after tissue injury in diabetic cardiomyopathy where its expression is significantly increased (Rajesh et al., 2010). In the present study, the extent of endometrial fibrosis was verified in rat IUA model by Masson staining along with the RT-qPCR validation of Collagen I, of which expression is a prognostic molecule in response to tissue damage as fibrosis. Transforming growth factor-β (TGF-β) family mediate cell fate-determining activities during growth, tissue homeostasis and regeneration, and are key participants in oncogenesis, fibrotic diseases, immune dysfunction and various genetic disorders (Heldin et al., 2012; Meng et al., 2016; Su et al., 2020). Both these well-known markers of fibrosis indicate exceptional defibrosis capability of VitroGel-supported MenSCs.

Physiologically, inflammation is usually the development of a pattern after tissue damage and is a critical stage of healing after injury, divided into several processes, including alteration of tissue, exudation of the inflammatory cells, release of inflammatory factors, and proliferation of local cells (Eming et al., 2017). In rat IUA model, the combination of MenSCs and VitroGel deactivates the wound-induced expression of IL-2 and derepresses IL-10. Such observation not only suggests the role of inflammation in IUA repairment, but also indicates the safety of both involved materials in terms of autoimmune concerns.

In summary, we find that MenSCs combined with VitroGel impede the fibrosis and facilitate the wound repair in IUA via anti-inflammation and angiogenesis, and eventually restore the fertility by increasing the endometrial receptivity, superiorly to either component individually. Although the more detailed underlying mechanism remains to be further elucidated, and the trajectory of the injected material warrants to be monitored for safety measurement by future studies, the present study shines promising light on high effectiveness of the combined treatment against IUA.

## Data Availability

The original contributions presented in the study are included in the article/Supplementary Material, further inquiries can be directed to the corresponding authors.

## Supplementary material

SUPPLEMENTARY FIGURE S1
*In vivo* imaging was performed to track MenSCs after injection into rat uterus. The images were captured at day 0 **(A)**, day 4 **(B)**, and day 8 **(C)**.

## References

[B1] Alcayaga-Miranda F. Cuenca J. Luz-Crawford P. Aguila-Díaz C. Fernandez A. Figueroa F. E. (2015). Characterization of menstrual stem cells: angiogenic effect, migration and hematopoietic stem cell support in comparison with bone marrow mesenchymal stem cells. Stem Cell Res. Ther. 6, 32. 10.1186/s13287-015-0013-5 25889741 PMC4404686

[B2] Alvarado-Velez M. Enam S. F. Mehta N. Lyon J. G. LaPlaca M. C. Bellamkonda R. V. (2021). Immuno-suppressive hydrogels enhance allogeneic MSC survival after transplantation in the injured brain. Biomaterials 266, 120419. 10.1016/j.biomaterials.2020.120419 33038594

[B3] Aurich I. Mueller L. P. Aurich H. Luetzkendorf J. Tisljar K. Dollinger M. M. (2007). Functional integration of hepatocytes derived from human mesenchymal stem cells into mouse livers. Gut 56, 405–415. 10.1136/gut.2005.090050 16928726 PMC1856821

[B4] Cai Y. Wu F. Yu Y. Liu Y. Shao C. Gu H. (2019). Porous scaffolds from droplet microfluidics for prevention of intrauterine adhesion. Acta Biomater. 84, 222–230. 10.1016/j.actbio.2018.11.016 30476581

[B5] Cao X. Duan L. Hou H. Liu Y. Chen S. Zhang S. (2020). IGF-1C hydrogel improves the therapeutic effects of MSCs on colitis in mice through PGE2-mediated M2 macrophage polarization. Theranostics 10, 7697–7709. 10.7150/thno.45434 32685014 PMC7359093

[B6] Chang Q. Y. Zhang S. W. Li P. P. Yuan Z. W. Tan J. C. (2020). Safety of menstrual blood-derived stromal cell transplantation in treatment of intrauterine adhesion. World J. Stem Cells 12, 368–380. 10.4252/wjsc.v12.i5.368 32547685 PMC7280865

[B7] Chaudhuri O. Klumpers D. Darnell M. Bencherif S. A. Weaver J. C. (2016). Hydrogels with tunable stress relaxation regulate stem cell fate and activity. Nat. Mater 15, 326–334. 10.1038/nmat4489 26618884 PMC4767627

[B8] Chen M. Xie L. (2016). Clinical evaluation of balloon occlusion of the lower abdominal aorta in patients with placenta previa and previous cesarean section: a retrospective study on 43 cases. Int. J. Surg. 34, 6–9. 10.1016/j.ijsu.2016.08.016 27545958

[B9] Chen Y.-R. Yan X. Yuan F. Lin L. Wang S. Ye J. (2022). Kartogenin-conjugated double-network hydrogel combined with stem cell transplantation and tracing for cartilage repair. Adv. Sci. (Weinh) 9, e2105571. 10.1002/advs.202105571 36253092 PMC9762312

[B10] de Almeida L. G. N. Thode H. Eslambolchi Y. Chopra S. Young D. Gill S. (2022). Matrix metalloproteinases: from molecular mechanisms to physiology, pathophysiology, and pharmacology. Pharmacol. Rev. 74, 714–770. 10.1124/pharmrev.121.000349 35738680

[B11] Deans R. Abbott J. (2010). Review of intrauterine adhesions. J. Minim. Invasive Gynecol. 17, 555–569. 10.1016/j.jmig.2010.04.016 20656564

[B12] Ebrahim N. Mostafa O. El Dosoky R. E. Ahmed I. A. Saad A. S. Mostafa A. (2018). Human mesenchymal stem cell-derived extracellular vesicles/estrogen combined therapy safely ameliorates experimentally induced intrauterine adhesions in a female rat model. Stem Cell Res. Ther. 9, 175. 10.1186/s13287-018-0924-z 29954457 PMC6027762

[B13] Eming S. A. Wynn T. A. Martin P. (2017). Inflammation and metabolism in tissue repair and regeneration. Science 356, 1026–1030. 10.1126/science.aam7928 28596335

[B14] Evans-Hoeker E. A. Young S. L. (2014). Endometrial receptivity and intrauterine adhesive disease. Semin. Reprod. Med. 32, 392–401. 10.1055/s-0034-1376358 24959821

[B15] Galipeau J. Sensebe L. (2018). Mesenchymal stromal cells: clinical challenges and therapeutic opportunities. Cell Stem Cell 22, 824–833. 10.1016/j.stem.2018.05.004 29859173 PMC6434696

[B16] Gao L. R. Chen Y. Zhang N. K. Yang X. L. Liu H. L. Wang Z. G. (2015). Intracoronary infusion of Wharton's jelly-derived mesenchymal stem cells in acute myocardial infarction: double-blind, randomized controlled trial. BMC Med. 13, 162. 10.1186/s12916-015-0399-z 26162993 PMC4499169

[B17] Gomaa Y. Kolluru C. Milewski M. Lee D. Zhang J. Saklatvala R. (2021). Development of a thermostable oxytocin microneedle patch. J. Control Release 337, 81–89. 10.1016/j.jconrel.2021.07.011 34265331

[B18] Gu L. H. Zhang T. T. Li Y. Yan H. J. Qi H. Li F. R. (2015). Immunogenicity of allogeneic mesenchymal stem cells transplanted via different routes in diabetic rats. Cell Mol. Immunol. 12, 444–455. 10.1038/cmi.2014.70 25242276 PMC4496541

[B19] Hamerlynck T. W. Blikkendaal M. D. Schoot B. C. Hanstede M. M. Jansen F. W. (2013). An alternative approach for removal of placental remnants: hysteroscopic morcellation. J. Minim. Invasive Gynecol. 20, 796–802. 10.1016/j.jmig.2013.04.024 24183271

[B20] Han M. Yang H. Lu X. Li Y. Liu Z. Li F. (2022). Three-Dimensional-cultured MSC-derived exosome-hydrogel hybrid microneedle array patch for spinal cord repair. Nano Lett. 22, 6391–6401. 10.1021/acs.nanolett.2c02259 35876503

[B21] Hare J. M. Fishman J. E. Gerstenblith G. DiFede Velazquez D. L. Zambrano J. P. Suncion V. Y. (2012). Comparison of allogeneic vs autologous bone marrow–derived mesenchymal stem cells delivered by transendocardial injection in patients with ischemic cardiomyopathy: the POSEIDON randomized trial. JAMA 308, 2369–2379. 10.1001/jama.2012.25321 23117550 PMC4762261

[B22] Hasani-Sadrabadi M. M. Sarrion P. Pouraghaei S. Chau Y. Ansari S. Li S. (2020). An engineered cell-laden adhesive hydrogel promotes craniofacial bone tissue regeneration in rats. Sci. Transl. Med. 12, eaay6853. 10.1126/scitranslmed.aay6853 32161103

[B23] He J. Zhang N. Zhu Y. Jin R. Wu F. (2021). MSC spheroids-loaded collagen hydrogels simultaneously promote neuronal differentiation and suppress inflammatory reaction through PI3K-Akt signaling pathway. Biomaterials 265, 120448. 10.1016/j.biomaterials.2020.120448 33068892

[B24] He W. Zhang X. Li X. Mao T. Lu Y. (2022). A decellularized spinal cord extracellular matrix-gel/GelMA hydrogel three-dimensional composite scaffold promotes recovery from spinal cord injury via synergism with human menstrual blood-derived stem cells. J. Mater Chem. B 10, 5753–5764. 10.1039/d2tb00792d 35838078

[B25] Heldin C.-H. Vanlandewijck M. Moustakas A. (2012). Regulation of EMT by TGFβ in cancer. FEBS Lett. 586, 1959–1970. 10.1016/j.febslet.2012.02.037 22710176

[B26] Hooker A. B. Lemmers M. Thurkow A. L. Heymans M. W. Opmeer B. C. Brolmann H. A. M. (2014). Systematic review and meta-analysis of intrauterine adhesions after miscarriage: prevalence, risk factors and long-term reproductive outcome. Hum. Reprod. Update 20, 262–278. 10.1093/humupd/dmt045 24082042

[B27] Ji W. Hou B. Lin W. Wang L. Zheng W. Li W. (2020). 3D Bioprinting a human iPSC-derived MSC-loaded scaffold for repair of the uterine endometrium. Acta Biomater. 116, 268–284. 10.1016/j.actbio.2020.09.012 32911103

[B28] Jin S. Choi H. Seong D. You C. L. Kang J. S. Rho S. (2023). Injectable tissue prosthesis for instantaneous closed-loop rehabilitation. Nature 623, 58–65. 10.1038/s41586-023-06628-x 37914945

[B29] Jirigala E. Yao B. Li Z. Zhang Y. J. Zhang C. Liang L. T. (2023). *In situ* forming injectable MSC-loaded GelMA hydrogels combined with PD for vascularized sweat gland regeneration. Mil. Med. Res. 10, 17. 10.1186/s40779-023-00456-w 37095579 PMC10127362

[B30] Khoury M. Alcayaga-Miranda F. Illanes S. E. Figueroa F. E. (2014). The promising potential of menstrual stem cells for antenatal diagnosis and cell therapy. Front. Immunol. 5, 205. 10.3389/fimmu.2014.00205 24904569 PMC4032935

[B31] Kuroda K. Yamanaka A. Takamizawa S. Nakao K. Kuribayashi Y. Nakagawa K. (2022). Prevalence of and risk factors for chronic endometritis in patients with intrauterine disorders after hysteroscopic surgery. Fertil. Steril. 118, 568–575. 10.1016/j.fertnstert.2022.05.029 35718544

[B32] Kwon M. Y. Vega S. L. Gramlich W. M. Kim M. Mauck R. L. Burdick J. A. (2018). Dose and timing of N-cadherin mimetic peptides regulate MSC chondrogenesis within hydrogels. Adv. Healthc. Mater 7, e1701199. 10.1002/adhm.201701199 29359863 PMC6296766

[B33] Lazarus H. M. Haynesworth S. E. Gerson S. L. Rosenthal N. S. Caplan A. I. (1995). *Ex vivo* expansion and subsequent infusion of human bone marrow-derived stromal progenitor cells (mesenchymal progenitor cells): implications for therapeutic use. Bone Marrow Transpl. 16, 557–564. 8528172

[B34] Li L. Xiao B. Mu J. Zhang Y. Zhang C. Cao H. (2019). A MnO2 nanoparticle-dotted hydrogel promotes spinal cord repair via regulating reactive oxygen species microenvironment and synergizing with mesenchymal stem cells. ACS Nano 13, 14283–14293. 10.1021/acsnano.9b07598 31769966

[B35] Li X.-C. Hao D.-Z. Hao W.-J. Guo X.-L. Jiang L. (2020). Bioinspired hydrogel-polymer hybrids with a tough and antifatigue interface via one-step polymerization. ACS Appl. Mater Interfaces 12, 51036–51043. 10.1021/acsami.0c14728 33112597

[B36] Li Y. Gao H. Brunner T. M. Hu X. Yan Y. Liu Y. (2022). Menstrual blood-derived mesenchymal stromal cells efficiently ameliorate experimental autoimmune encephalomyelitis by inhibiting T cell activation in mice. Stem Cell Res. Ther. 13, 155. 10.1186/s13287-022-02838-8 35410627 PMC8995916

[B37] Lindsey M. L. (2018). Assigning matrix metalloproteinase roles in ischaemic cardiac remodelling. Nat. Rev. Cardiol. 15, 471–479. 10.1038/s41569-018-0022-z 29752454 PMC6203614

[B38] Liu F. Hu S. Yang H. Li Z. Huang K. Su T. (2019). Hyaluronic acid hydrogel integrated with mesenchymal stem cell-secretome to treat endometrial injury in a rat model of Asherman's syndrome. Adv. Healthc. Mater 8, e1900411. 10.1002/adhm.201900411 31148407 PMC7045702

[B39] Mazzon I. Favilli A. Cocco P. Grasso M. Horvath S. Bini V. (2014). Does cold loop hysteroscopic myomectomy reduce intrauterine adhesions? A retrospective study. Fertil. Steril. 101, 294–298.e3. 10.1016/j.fertnstert.2013.09.032 24182410

[B40] Meng X.-m. Nikolic-Paterson D. J. Lan H. Y. (2016). TGF-β: the master regulator of fibrosis. Nat. Rev. Nephrol. 12, 325–338. 10.1038/nrneph.2016.48 27108839

[B41] Mirzadegan E. Golshahi H. Saffarian Z. Darzi M. Khorasani S. Edalatkhah H. (2022). The remarkable effect of menstrual blood stem cells seeded on bilayer scaffold composed of amniotic membrane and silk fibroin aiming to promote wound healing in diabetic mice. Int. Immunopharmacol. 102, 108404. 10.1016/j.intimp.2021.108404 34863653

[B42] Pabuccu R. Onalan G. Kaya C. Selam B. Ceyhan T. Ornek T. (2008). Efficiency and pregnancy outcome of serial intrauterine device–guided hysteroscopic adhesiolysis of intrauterine synechiae. Fertil. Steril. 90, 1973–1977. 10.1016/j.fertnstert.2007.06.074 18774563

[B43] Perez-Medina T. Bajo-Arenas J. Salazar F. Redondo T. Sanfrutos L. Alvarez P. (2005). Endometrial polyps and their implication in the pregnancy rates of patients undergoing intrauterine insemination: a prospective, randomized study. Hum. Reprod. 20, 1632–1635. 10.1093/humrep/deh822 15760959

[B44] Phinney D. G. Galipeau J. Krampera M. Martin I. Shi Y. Sensebe L. (2013). MSCs: science and trials. Nat. Med. 19, 812. 10.1038/nm.3220 23836216

[B45] Qi J. Zhang X. Zhang S. Wu S. Lu Y. Li S. (2023). P65 mediated UBR4 in exosomes derived from menstrual blood stromal cells to reduce endometrial fibrosis by regulating YAP Ubiquitination. J. Nanobiotechnology 21, 305. 10.1186/s12951-023-02070-3 37644565 PMC10463480

[B46] Qiao Z. Lian M. Han Y. Sun B. Zhang X. Jiang W. (2021). Bioinspired stratified electrowritten fiber-reinforced hydrogel constructs with layer-specific induction capacity for functional osteochondral regeneration. Biomaterials 266, 120385. 10.1016/j.biomaterials.2020.120385 33120203

[B47] Rajesh M. Mukhopadhyay P. Bátkai S. Patel V. Saito K. Matsumoto S. (2010). Cannabidiol attenuates cardiac dysfunction, oxidative stress, fibrosis, and inflammatory and cell death signaling pathways in diabetic cardiomyopathy. J. Am. Coll. Cardiol. 56, 2115–2125. 10.1016/j.jacc.2010.07.033 21144973 PMC3026637

[B48] Shen K. Sun G. Chan L. He L. Li X. Yang S. (2021). Anti-inflammatory nanotherapeutics by targeting matrix metalloproteinases for immunotherapy of spinal cord injury. Small 17, e2102102. 10.1002/smll.202102102 34510724

[B49] Su J. Morgani S. M. David C. J. Wang Q. Er E. E. Huang Y. H. (2020). TGF-β orchestrates fibrogenic and developmental EMTs via the RAS effector RREB1. Nature 577, 566–571. 10.1038/s41586-019-1897-5 31915377 PMC7450666

[B50] Tan J. Li P. Wang Q. Li Y. Li X. Zhao D. (2016). Autologous menstrual blood-derived stromal cells transplantation for severe Asherman's syndrome. Hum. Reprod. 31, 2723–2729. 10.1093/humrep/dew235 27664218

[B51] Tang Q. Lu B. He J. Chen X. Fu Q. Han H. (2022). Exosomes-loaded thermosensitive hydrogels for corneal epithelium and stroma regeneration. Biomaterials 280, 121320. 10.1016/j.biomaterials.2021.121320 34923312

[B52] Uccelli A. Moretta L. Pistoia V. (2008). Mesenchymal stem cells in health and disease. Nat. Rev. Immunol. 8, 726–736. 10.1038/nri2395 19172693

[B53] Wan C. He Q. McCaigue M. Marsh D. Li G. (2006). Nonadherent cell population of human marrow culture is a complementary source of mesenchymal stem cells (MSCs). J. Orthop. Res. 24, 21–28. 10.1002/jor.20023 16419965

[B54] Wang L. Yu C. Chang T. Zhang M. Song S. Xiong C. (2020). *In situ* repair abilities of human umbilical cord-derived mesenchymal stem cells and autocrosslinked hyaluronic acid gel complex in rhesus monkeys with intrauterine adhesion. Sci. Adv. 6, eaba6357. 10.1126/sciadv.aba6357 32494750 PMC7244313

[B55] Wang Y. Zhao Y. Ge Y. Cen J. Liao Y. Xu G. (2022). Reproductive outcomes and reproductive tract microbiota shift in women with moderate-to-severe intrauterine adhesions following 30-day post-hysteroscopic placement of balloon stents or intrauterine contraceptive devices: a randomized controlled trial. EClinicalMedicine 43, 101200. 10.1016/j.eclinm.2021.101200 35128361 PMC8808160

[B56] Wechsler M. E. Rao V. V. Borelli A. N. Anseth K. S. (2021). Engineering the MSC secretome: a hydrogel focused approach. Adv. Healthc. Mater 10, e2001948. 10.1002/adhm.202001948 33594836 PMC8035320

[B57] Wisdom K. M. Adebowale K. Chang J. Lee J. Y. Nam S. Desai R. (2018). Matrix mechanical plasticity regulates cancer cell migration through confining microenvironments. Nat. Commun. 9, 4144. 10.1038/s41467-018-06641-z 30297715 PMC6175826

[B58] Xin L. Zheng X. Chen J. Hu S. Luo Y. Ge Q. (2022). An acellular scaffold facilitates endometrial regeneration and fertility restoration via recruiting endogenous mesenchymal stem cells. Adv. Healthc. Mater 11, e2201680. 10.1002/adhm.202201680 36049781

[B59] Xu B. Zhou M. Liu M. Wang Z. Duan J. Li W. (2023). Bioactive injectable and self-healing hydrogel via cell-free fat extract for endometrial regeneration. Small 19, e2300481. 10.1002/smll.202300481 37035992

[B60] Yu D. Wong Y.-M. Cheong Y. Xia E. Li T.-C. (2008). Asherman syndrome--one century later. Fertil. Steril. 89, 759–779. 10.1016/j.fertnstert.2008.02.096 18406834

[B61] Zhang S. Huang B. Su P. Chang Q. Li P. Song A. (2021). Concentrated exosomes from menstrual blood-derived stromal cells improves ovarian activity in a rat model of premature ovarian insufficiency. Stem Cell Res. Ther. 12, 178. 10.1186/s13287-021-02255-3 33712079 PMC7953711

[B62] Zhang S. Li P. Yuan Z. Tan J. (2019). Platelet-rich plasma improves therapeutic effects of menstrual blood-derived stromal cells in rat model of intrauterine adhesion. Stem Cell Res. Ther. 10, 61. 10.1186/s13287-019-1155-7 30770774 PMC6377773

[B63] Zhang X. Chen G. Wang Y. Fan L. Zhao Y. (2022). Arrowhead composite microneedle patches with anisotropic surface adhesion for preventing intrauterine adhesions. Adv. Sci. (Weinh) 9, e2104883. 10.1002/advs.202104883 35187857 PMC9036003

[B64] Zheng F. Xin X. He F. Liu J. Cui Y. (2020). Meta-analysis on the use of hyaluronic acid gel to prevent intrauterine adhesion after intrauterine operations. Exp. Ther. Med. 19, 2672–2678. 10.3892/etm.2020.8483 32256748 PMC7086218

[B65] Zheng S. X. Wang J. Wang X. Ali A. Wu L. Liu Y. (2018). Feasibility analysis of treating severe intrauterine adhesions by transplanting menstrual blood-derived stem cells. Int. J. Mol. Med. 41, 2201–2212. 10.3892/ijmm.2018.3415 29393381

[B66] Zinter M. S. Delucchi K. L. Kong M. Y. Orwoll B. E. Spicer A. S. Lim M. J. (2019). Early plasma matrix metalloproteinase profiles. A novel pathway in pediatric acute respiratory distress syndrome. Am. J. Respir. Crit. Care Med. 199, 181–189. 10.1164/rccm.201804-0678OC 30114376 PMC6353006

