# Supplementary figures and images for: VitroGel-loaded human MenSCs promote endometrial regeneration and fertility restoration
Source: Front Bioeng Biotechnol. 2024 Jan 8;11:1310149. doi: 10.3389/fbioe.2023.1310149 (PMC10800509; doi:10.3389/fbioe.2023.1310149)

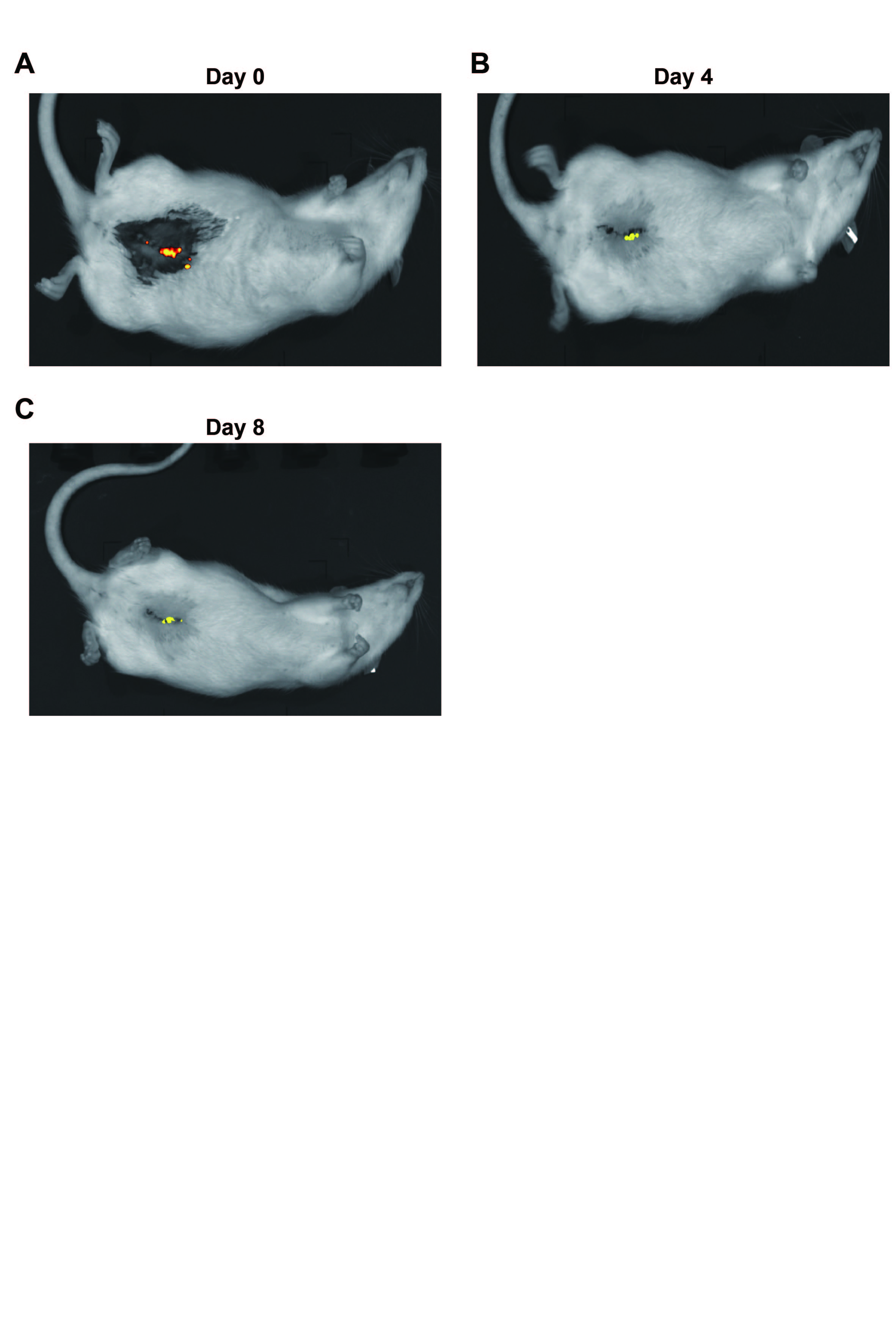

Supplement: Supplementary file 1 [file Image1.JPEG]
